# A Map of the Lipid–Metabolite–Protein Network to Aid Multi-Omics Integration

**DOI:** 10.3390/biom15040484

**Published:** 2025-03-26

**Authors:** Uchenna Alex Anyaegbunam, Aimilia-Christina Vagiona, Vincent ten Cate, Katrin Bauer, Thierry Schmidlin, Ute Distler, Stefan Tenzer, Elisa Araldi, Laura Bindila, Philipp Wild, Miguel A. Andrade-Navarro

**Affiliations:** 1 Computational Biology and Data Mining Group (CBDM), Institute of Organismic and Molecular Evolution (iOME), Johannes Gutenberg University, 55122 Mainz, Germany; 2 Preventive Cardiology and Preventive Medicine, Department of Cardiology, University Medical Center, Johannes-Gutenberg University Mainz, Langenbeckstr. 1, 55131 Mainz, Germany; 3 Clinical Epidemiology and Systems Medicine, Center for Thrombosis and Hemostasis (CTH), University Medical Center, 55131 Mainz, Germany; 4 German Center for Cardiovascular Research (DZHK), Partner Site Rhine Main, University Medical Center, Johannes-Gutenberg University Mainz, 55131 Mainz, Germany; 5 Computational Systems Medicine, Center for Thrombosis and Hemostasis (CTH), 55131 Mainz, Germany; 6 Institute of Immunology, University Medical Center, Johannes-Gutenberg University Mainz, 55131 Mainz, Germany; 7 Research Centre for Immunotherapy (FZI), University Medical Center, Johannes-Gutenberg University Mainz, 55131 Mainz, Germany; 8 Systems Medicine Laboratory, Department of Medicine and Surgery, University of Parma, 43121 Parma, Italy; 9 Institute of Physiological Chemistry, University Medical Center, 55131 Mainz, Germany

**Keywords:** lipids, metabolites, proteins, cardiovascular diseases, multi-omics integration, lipid–metabolite–protein network, omics

## Abstract

The integration of multi-omics data offers transformative potential for elucidating complex molecular mechanisms underlying biological processes and diseases. In this study, we developed a lipid–metabolite–protein network that combines a protein–protein interaction network and enzymatic and genetic interactions of proteins with metabolites and lipids to provide a unified framework for multi-omics integration. Using hyperbolic embedding, the network visualizes connections across omics layers, accessible through a user-friendly Shiny R (version 1.10.0) software package. This framework ranks molecules across omics layers based on functional proximity, enabling intuitive exploration. Application in a cardiovascular disease (CVD) case study identified lipids and metabolites associated with CVD-related proteins. The analysis confirmed known associations, like cholesterol esters and sphingomyelin, and highlighted potential novel biomarkers, such as 4-imidazoleacetate and indoleacetaldehyde. Furthermore, we used the network to analyze empagliflozin’s temporal effects on lipid metabolism. Functional enrichment analysis of proteins associated with lipid signatures revealed dynamic shifts in biological processes, with early effects impacting phospholipid metabolism and long-term effects affecting sphingolipid biosynthesis. Our framework offers a versatile tool for hypothesis generation, functional analysis, and biomarker discovery. By bridging molecular layers, this approach advances our understanding of disease mechanisms and therapeutic effects, with broad applications in computational biology and precision medicine.

## 1. Introduction

The integration of multi-omics data offers immense potential for discovering intricate molecular mechanisms in biological systems [1,2]. For example, combining lipidomics and transcriptomics has identified critical interactions in prostate cancer, such as the specificity of sphingosine in distinguishing cancerous from benign conditions and its downstream signaling implications [3,4]. Similarly, the integration of proteomics with genomic and transcriptomic data has been instrumental in pinpointing potential cancer driver genes and their pathways, as shown in colon cancer studies [1,5]. However, practical challenges remain, including the heterogeneity of data types, variations in data quality, and the high dimensionality of omics datasets, which complicate data alignment and biological interpretation [6]. These challenges hinder the merging of diverse omics layers—such as proteomics, lipidomics, and metabolomics—into a unified framework [6,7,8,9]. Specific examples include difficulties in aligning multi-omics data due to different scales and formats, and limitations in the interpretability of integrated models when applied to toxicological studies [2,6]. Furthermore, the lack of standardization in experimental protocols and data preprocessing often results in sparsely connected datasets with reduced biological coherence [7].

These issues have been addressed with various strategies, such as early integration and hierarchical integration approaches, deep learning methods, and network-based frameworks [10,11,12,13,14,15,16,17,18,19,20,21,22,23,24,25,26,27,28,29,30,31]. For instance, early integration models combine omics data into a single representation, enabling the simultaneous analysis and identification of cross-modal patterns. Frameworks such as those proposed by [10,11] directly combine data from different omics layers, thereby enhancing predictive accuracy for tasks like biomarker discovery—a strategy comprehensively reviewed by [12]. Hierarchical integration models leverage the strengths of individual layers before integrating them in a stepwise manner to enhance interpretability and predictive power [12]. This approach, exemplified by dual-path graph attention auto-encoders [13] and unsupervised neural networks, like UMINT [14], preserves spatial or single-cell resolution while mitigating issues related to data sparsity and high dimensionality. Complementary frameworks, such as MUON [15], further refine this multi-tiered analysis. Deep learning approaches leverage adaptive training to each omics layer independently and transformer architectures to optimize cross-modality interaction learning to maximize data utility. Methods like CustOmics [16] and scmFormer [17] have been successfully applied to large-scale proteomic–transcriptomic integration. These approaches have not only improved predictive performance in complex phenotypes—as highlighted in studies on neurodegenerative diseases [18] and multimodal deep learning reviews [19]—but have also benefited from transfer learning strategies exemplified by scJoint [20]. Additionally, scCross [21] addresses omics integration problems by overcoming modality discrepancies, data scarcity in less robust modalities, and complex cross-modal alignment challenges, thereby enabling effective cross-modal data generation, simulation, and in silico cellular perturbation analysis.

Network-based frameworks exploit graph-theoretical models to capture the intricate interdependencies among molecular entities. Graph-linked embedding techniques, like GLUE [22], facilitate regulatory inference from single-cell data, while graph convolutional networks employed in MoGCN [23] offer refined analysis for cancer subtype classification. Hypergraph integration networks, such as MORE [24], enhance biomedical classification and biomarker identification, and executable network models [25] provide dynamic platforms for simulating multi-omics interactions. Additional frameworks, like TEMINET [26] and scBridge [27], further address cellular heterogeneity. TEMINET builds disease-specific networks from intra-omics features and then leverages graph attention networks within a multi-level framework to capture comprehensive collective information beyond simple pairwise interactions, while scBridge iteratively selects scATAC-seq cells with minimal omics differences and integrates them with scRNA-seq.

In this study, we present a network embedded in hyperbolic space that integrates human lipids, metabolites, and proteins to aid multi-omics integrative research requiring the interpretation of the associations among these three particular molecular types. Accordingly, the nodes of the network are proteins, lipids, and metabolites. Edges connect proteins with interacting proteins, metabolites, and lipids. There are several algorithms and models supporting that the topology of complex systems, such as interactomes, is shaped by an underlying hidden hyperbolic geometry [32,33]. Modeling complex systems in hyperbolic geometry offers a novel way to analyze their hierarchical and clustering properties, which is more appropriate for densely connected networks and has implications for understanding how nodes interact and how disease effects propagate in networks [34,35].

To facilitate multi-omics integration, this network is used as a backend database for software implementation that considers three molecular types: proteins, lipids, and metabolites. Using this tool, a user can input a set of molecules of one of the three molecular types (for example, dysregulated proteins or proteins with a common biological function) and retrieve a ranked list of the other two molecular types (for example, lipids and metabolites). First, we demonstrate the utility of the tool for literature discovery from an omics layer in the context of studying a disease. Here, we focus on cardiovascular diseases, a major area of interest due to the complexity and interrelationships of lipid and metabolic dysregulation involved [36,37,38,39,40]. Secondly, we show how our method offers opportunities for functional enrichment analysis of lipidomic profiles.

## 2. Methods

### 2.1. Construction of a Protein, Lipid, and Metabolite Network

We started from the protein–protein interaction network of the HIPPIE database (version 2.3) [41]. This database assigns confidence scores (from 0, low, to 1, high) to interactions between human proteins based on the type and amount of experimental information available in biomedical publications [42]. In this study, only protein–protein interactions with a confidence score of ≥0.71 were kept (following [34]) because this selects a high percentage of interactions supported by at least two publications. The network’s largest connected component (subset of connected nodes) was obtained. This consisted of 15,412 nodes (proteins) and 185,793 edges. Proteins not connected to this subset are not mappable by definition and were ignored.

Lipids (detectable in human plasma, as defined in [43]) related to the proteins in this initial network were then added using information from enzymatic reactions sourced from the SwissLipid database (967 interactions, [44]), and links from lipids to gene loci from a GWAS (652 interactions, [45]). This resulted in the addition of 940 lipids to the network.

In addition, metabolites (detectable in human plasma, according to [46]) related to proteins were then added using enzymatic reaction information from PubChem (1471 interactions, [47]). This resulted in the addition of 273 metabolites to the network. In total, the network consists of 16,625 nodes (Supplementary Table S1) and 188,883 edges (Supplementary Table S2).

### 2.2. Hyperbolic Embedding of the Network

To facilitate visual interpretation and to reflect the overall connectivity of the network, we applied hyperbolic mapping techniques. We embedded the multi-omics network in the two-dimensional hyperbolic plane using the R package “NetHypGeom” (version 1.0), which implements the LaBNE + HM algorithm [48]. This approach combines maximum likelihood estimation and manifold learning to decipher the underlying hyperbolic geometry of complex networks [49,50]. The popularity–similarity (PS) model provides a geometric interpretation in hyperbolic space (H^2^) and assumes that the clustering and hierarchy of complex networks arise from trade-offs between the popularity and similarity of nodes [51]. In this embedding, the hyperbolic distance reflects their similarity, such that closer pairs of nodes in the hyperbolic space are more likely to have connections in the network [50,51,52]. The network was embedded in H^2^ to infer the hyperbolic coordinates of each node, with parameters γ = 2.98, T = 0.84, and w = 2π. In the embedding, the 16,625 nodes of the multi-omics network lie within a hyperbolic disk where the radial coordinate of a node, *r_i_*, represents the popularity dimension with nodes that joined the system first being close to the disk’s center. The angular coordinate of a node, *θ_i_*, represents the similarity dimension. The hyperbolic coordinates (*r* and *θ*) were used to compute the hyperbolic distance between nodes in the hyperbolic map. This representation can be interpreted as molecular relationships within the context of specific biological processes.

### 2.3. Clustering in the Angular Similarity Dimension

To cluster nodes in the similarity dimension, we computed the difference between consecutive angular coordinates to identify gaps. The nodes were sorted according to their inferred angles *θ* in increasing order, and the difference between *θ_i_* and *θ_i_*_+1_ was computed to identify the largest gaps between clusters in the similarity dimension. To determine the start and the end of each cluster, we chose gap sizes that produced clusters with a minimum number of 10 protein members because this allowed us to perform meaningful functional enrichment analysis of each sector.

### 2.4. Evaluation of Molecular Relationships Between Omics Layers

The hyperbolic network offers the possibility of measuring distances (in hyperbolic space) among all nodes (proteins, lipids, and metabolites), which can be interpreted as functional relationships. We implemented a user-friendly Shiny R (version 1.10.0) software package ((https://github.com/uchealex/Omint) (accessed on 24 March 2025)) that ranks molecules between omics layers based on their hyperbolic distances. The method uses as input a dataset of molecules of one of the three molecular types (for example, a list of differentially expressed proteins), and provides a list of all of the molecules in each of the other two molecular types (for example, lipids or metabolites), ranked according to their distances to the input dataset.

The ranking score is generated by taking the reciprocal of the sum of the n smallest distances (n = 3, by default) between the nodes corresponding to the user-defined subset (for example, proteins) and the nodes of the other two molecular types (for example, lipids or metabolites). Therefore, higher scores indicate stronger associations between the nodes of the subset and the nodes of the other molecular types. The algorithmic implementation of our method is based on the pseudo-code shown in Box 1.

Box 1 **Algorithm for cross-omics association ranking using hyperbolic geometry. This schematic outlines the computational workflow for identifying molecular associations between omics layers. The algorithm (1) processes user-input molecular identifiers of one omics layer, (2) calculates hyperbolic distances between all valid input molecules and all items in the other (non-input) layers, (3) computes association scores as the reciprocal sum of the n smallest distances to the items in each of the non-input layers (default n = 3), and (4) generates ranked lists of associated molecules in the non-input layers with evidence trails.**

Algorithm: Cross-omics Relationship Ranking via Hyperbolic Distance

Input:

- User-defined molecule subset (S): List of identifiers of one omics layer (proteins, lipids, or metabolites)
- Hyperbolic coordinates dataset: Precomputed (r, θ) for all molecules across layers
- n: Number of smallest distances to consider (default = 3)

Output:

- Ranked lists of molecules of non-input omics layers, sorted by association strength

Procedure:

1. Input Processing:
  - Receive user input (S) and parameter n
  - Validate identifiers in S against reference database
  - Filter valid subset (S_valid) = S ∩ database_entries
2. Distance Matrix Construction:
  For each target omics layer T ∉ input layer:
  - Initialize distance matrix D with dimensions |S_valid| × |T|
  - Compute hyperbolic distances between all pairs (s ∈ S_valid, t ∈ T):
    distance(s,t) = acosh[cosh(r_s_)cosh(r_t_) − sinh(r_s_)sinh(r_t_)cos(Δθ)]
    where Δθ = π − |π − |θ_s_ − θ_t_||
3. Association Score Calculation:
  For each molecule t in target layer T:
  - Collect all distances from S_valid to t: {distance(s_1_,t), …, distance(s_k_,t)}
  - Identify n smallest distances: d_1_ ≤ d_2_ ≤ … ≤ d_n_
  - Compute association score: score(t) = 1/(Σ^n^_{i=1}_d_i_)
4. Ranking and Output:
  - Sort all molecules in T by descending score(t)
  - Generate evidence strings for top associations:
    evidence(t) = [si:di (sorted)] for i = 1…n
  - Return ranked list: (t, score(t), evidence(t)) ∀ t ∈ T

### 2.5. Functional Enrichment Analyses

We carried out Gene Ontology (GO) enrichment analysis for the proteins in each sector of the multi-omics network, using the proteins of the complete network as a background set. For functional enrichment analysis, we used the Enrichr R Package (version 3.4). Enrichr is a comprehensive tool that provides access to multiple gene set libraries and databases, enabling the identification of significantly enriched biological pathways and functional terms [53]. Only GO biological process (BP) terms enriched at a significance level (*p*-value, multiple testing corrected) of 0.05 or less were retained. A similar methodology was used to compute enrichment analysis for sets of proteins associated with empagliflozin treatment.

## 3. Results

To provide a holistic view of molecular interactions across multiple omics layers, including lipids, metabolites, and proteins, we constructed a network by combining public data describing experimental interactions among them. The network was embedded in hyperbolic space (see Section 2 for details). By using hyperbolic mapping, we enable researchers to explore molecular relationships in a visually intuitive and biologically meaningful way.

**Figure 1:**
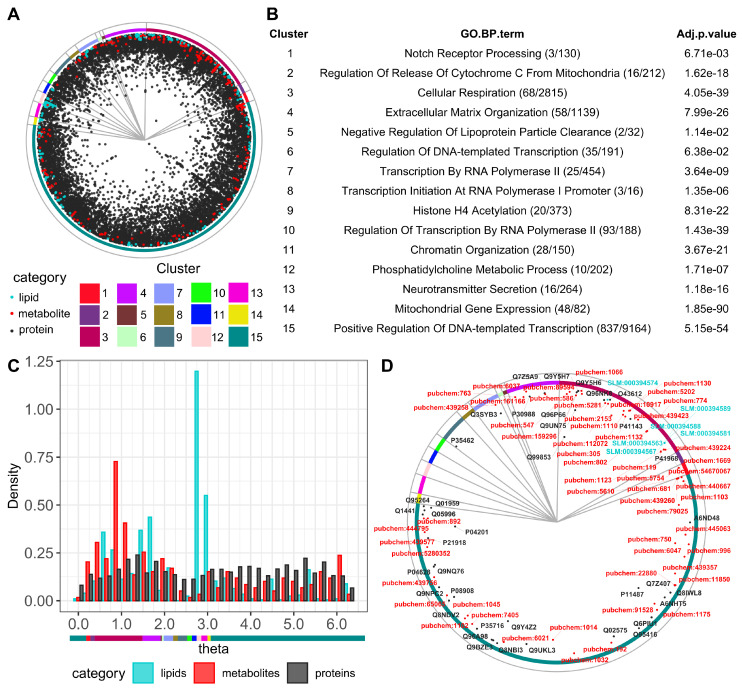
Visualization and functional characterization of the human protein–lipid–metabolite interaction network. (**A**) Network integration of multi-omics data embedded in the hyperbolic space (see Section 2 for details). Node colors represent different molecular classes (proteins in black, lipids in cyan, metabolites in red). The circular sectors indicate clusters (numbered 1 to 15) identified by the gaps between consecutive nodes in the angular dimension of the hyperbolic space (see Section 2 for details). Node coordinates are available in Supplementary Table S1. Edges (not represented) are available in Supplementary Table S2). (**B**) The over-represented biological function in each cluster was determined via GO enrichment analysis (BP: biological process) to reveal the biological relevance based on the term with the lowest adjusted *p*-value of enrichment (see Section 2 for details). The numbers in parentheses indicate how many proteins had the indicated GO term out of all the proteins in the respective cluster. (**C**) The distribution of *θ* coordinates for each omics layer: proteins, lipids, and metabolites. The different maxima positions of lipids and metabolites in the angular dimension indicate that they are grouped in different clusters. The angular coordinates of the 15 clusters are indicated with a colored bar for reference under the plot. (**D**) Brain-related proteins, lipids, and metabolites in the hyperbolic map (identifiers from UniProt, SLM, and PubChem, respectively).

In hyperbolic space, nodes have polar coordinates (*r*, *θ*). Nodes with shorter distances to the center (low *r* values) correspond to nodes with higher connectivity. The similarity in the *θ* coordinates between nodes reflects their similar interactions with other nodes [32,34,35]. To exploit the biological meaning of the *θ* coordinates in the multi-omics network, we clustered the elements of the network into groups based on the identified gaps between consecutive angles and determined 15 clusters (see Section 2 for details; Figure 1A). To evaluate the functions represented in each sector, we determined the enrichment in GO biological process terms in the proteins of each cluster (Figure 1B). The distribution of proteins, lipids, and metabolites in the angular dimension highlights how lipids and metabolites agglomerate in similarity-based clusters. For example, the angular distribution of lipids has a maximum of around *θ* = 2.8, which corresponds to cluster 12. Accordingly, cluster 12 is enriched in proteins annotated with the term Phosphatidylcholine Metabolic Process, indicating the biological significance of the similarity component of the map (Figure 1C). The angular distribution of metabolites has a maximum of around *θ* = 1.0 in cluster 3, which is enriched in proteins annotated with the term Cellular Respiration; this reflects the central role of cellular respiration in cellular metabolism (Figure 1C).

To test the coherence across omics layers, we annotated lipids (using SwissLipids [44]) and metabolites (using HMDB [54]) present in the brain and proteins specifically expressed in the brain (according to all brain tissues represented in GTEx [55]). This resulted in a selection of 6 lipids, 55 metabolites, and 99 proteins. Their distributions overlapped across clusters and were absent in a few clusters on the top left part of the map (Figure 1D). The lipids are grouped in the cellular respiration cluster, while proteins and metabolites are more widely distributed.

Interestingly, while cluster 13 is enriched in neurotransmitter secretion proteins, this cluster is devoid of brain-specific molecules. This reflects the fact that the machinery for vesicle fusion and regulated secretion uses proteins that are expressed in neural and non-neural tissues. For example, one of these proteins is Unc-13 homolog A (UNC13A, a.k.a. SYT14L). While this protein has neural functions and expression, and has been shown to extend dendrite length, it also regulates melanocyte differentiation [56]. Another example is synaptotagmin-1 (SYT1), which is well-known for neural functions but has also been associated with endocytosis in pancreatic beta-cells [57]. These observations indicate that our map can be used to obtain useful insight into biological function by leveraging known associations among proteins, lipids, and metabolites.

### 3.1. Software Implementation

**Figure 2:**
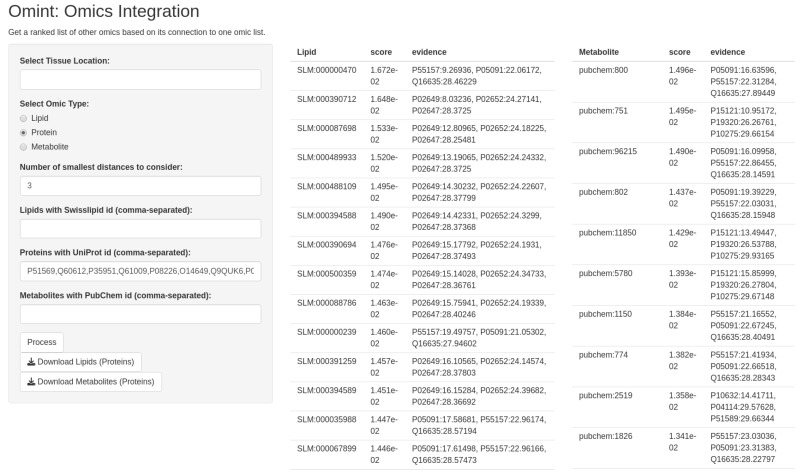
Software implementation for omics integration. Our method was implemented using the Shiny R framework to allow users to input a list of molecules of one type (proteins, lipids, or metabolites) and retrieve molecules of the other two molecular types ranked by their distances to the input subset in the hyperbolic map.

To take advantage of the possibilities that our map offers to exploit biological information connected across proteins, lipids, and metabolites, we developed a user-friendly software implementation that utilizes the Shiny R framework to allow users to input a list of molecules of one molecular type (subset) and retrieve ranked lists of the other two molecular types according to their distances to the input subset in the hyperbolic map (Figure 2). For instance, a user can input a list of proteins and retrieve ranked lipids or metabolites based on their proximity to the input proteins in the network (see Section 2 for details).

To use the software, the user first indicates what type of molecule is provided as the input. Secondly, the user chooses the number of closest molecules from the user-defined subset to be used to rank the other sets (the default number used here was three). Finally, the user inputs a list of molecules (subset) and clicks the “process” button to produce and display ranked lists of each of the other two molecular types. These lists can be downloaded by clicking the corresponding “download” buttons.

Optionally, the user can filter the analysis for proteins expressed in a selection of tissues. For this, we systematically annotated proteins for their messenger RNA (mRNA) tissue-specific expression using data from the Human Protein Atlas Database [58], considering expression profiles across 50 tissues. Specifically, each protein of the network was mapped to its corresponding tissue-specific RNA expression profile, setting a cut-off of 10 nTMP (normalized transcripts per million). This value is generally considered a moderate to high expression level, reducing false positive cases and improving the biological significance of tissue annotations.

The software we developed enhances the utility of our protein–lipid–metabolite network by allowing researchers to explore multi-omics connections, enabling hypothesis generation and the identification of potential biomarkers. In the next sections, we illustrate the use of the method with two use cases.

### 3.2. Literature Discovery: Cardiovascular Disease Case Study

**Figure 3:**
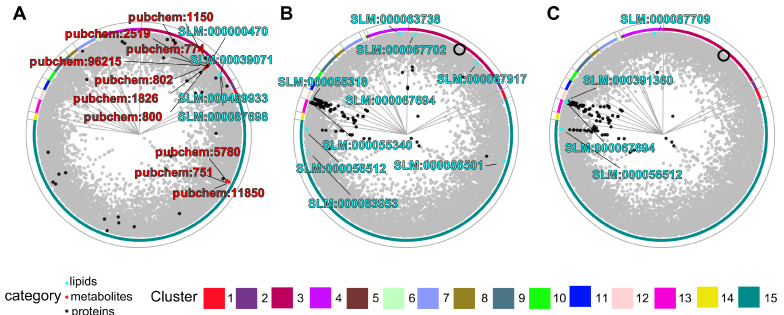
Application of the hyperbolic map to infer associations among molecular types. Different views of the human protein–lipid–metabolite interaction network (nodes in grey) highlighting (**A**) a subset of proteins related to the literature investigating lipids in CVD, used as input to score associated lipids (top four shown; cyan; see also Table 1; full data in Supplementary Table S4) and metabolites (top ten shown; red; see also Table 2; full data in Supplementary Table S5), and (**B**,**C**) subsets of lipids (cyan) that were dysregulated at two time points of empagliflozin treatment (one and twelve weeks, respectively), used as input to obtain related proteins (top 100 shown, black; full data in Supplementary Table S6; the black empty circle indicates the position of SGLT2, the protein inhibited by empagliflozin).

It is well known that cardiovascular diseases (CVDs) are accompanied by the dysregulation of certain proteins, lipids, and metabolites. To find lipids and metabolites with relevance to CVD, we used a list of proteins selected for their importance in the literature on lipid research in CVD by [43] (Supplementary Table S4) as input to our software to derive a ranked list of lipids and metabolites closely associated with these proteins (Supplementary Tables S5 and S6) (Figure 3A). In short, the proteins were selected for their enzymatic connections to lipids associated with CVD, as addressed in PubMed records with a focus on physiological research on humans and mice (mentioning plasma, heart, or myocardium).

The four top-ranked lipids (cholesterol esters, sphingomyelin, phosphatidylcholine, and ganglioside) had over 600 citations in publications relating to CVD (Table 1). The ten top-ranked metabolites had over 850 citations in publications in relation to CVD (Table 2). The metabolites with the most citations were caffeine and histamine. Altogether, our method is able to identify lipids and metabolites that have been previously implicated in pathways relevant to cardiovascular health. Furthermore, highly ranked metabolites with low literature presence, such as 4-imidazoleacetate and indoleacetaldehyde, could be unknown potential biomarkers of CVD.

### 3.3. Functional Enrichment Analysis of Lipid Signatures

Empagliflozin is an antidiabetic drug that works as an inhibitor of the sodium-glucose co-transporter-2 (SGLT2) [59]. Lipid signatures of treatment with empagliflozin at two time points (one week and twelve weeks after treatment) are available from the EmDia study, a placebo-controlled randomized phase 3 clinical study on the effect of empagliflozin on left ventricular diastolic function [60,61]. To add information on the drug’s temporal effects, we used as input to our tool the list of one- and twelve-week empagliflozin signature lipids present in our map (Supplementary Tables S7 and S8) to score human proteins for their association with them (Supplementary Tables S9 and S10). The map positions of the signature lipids and the top 100 scored proteins (cyan and black, respectively; Figure 3B,C) suggest a shift where, at the one-week time point, some dysregulated lipids (and some of the top scoring proteins) are located in the same or neighboring clusters as SGLT2 in the map (black circle, Figure 3B,C), whereas, at the twelve-week time point, most of the mapped dysregulated lipids are close to the clusters of the top-scoring proteins on the left of the map.

**Figure 4:**
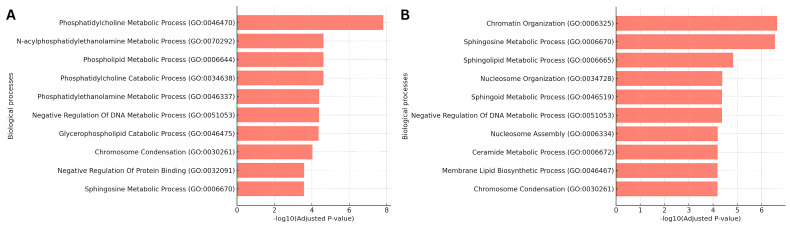
Temporal Gene Ontology (GO) enrichment analysis of lipid signatures following empagliflozin treatment. (**A**) Enriched GO biological processes for proteins associated with lipid signatures at one week and (**B**) twelve weeks after empagliflozin treatment, showing shifts from phospholipid metabolism to sphingolipid and ceramide metabolism. Functional enrichment was carried out using the top 100 proteins derived from lipid signatures. Full lists are given in Supplementary Tables S7 and S8, respectively.

To interpret this temporal shift in protein function, the top 100 proteins at each time point (Figure 3B,C) were used for GO enrichment analysis (Figure 4; Supplementary Tables S11 and S12). For the one-week time point, the primary enriched terms were phosphatidylcholine metabolic process, phosphatidylethanolamine metabolic process, and sphingosine metabolic process. These pathways highlight the initial biochemical shifts triggered by empagliflozin treatment, with early alterations emphasizing general phospholipid metabolism.

By twelve weeks, the enrichment profile shifted to emphasize the sphingosine metabolic process and the ceramide metabolic process. The sustained enrichment of sphingosine metabolism at both time points underscores its central role in the long-term effects of empagliflozin. This aligns with experimental findings in the EmDia study [60,61] showing an increase in sphingomyelins and ceramides, which are integral to sphingosine metabolism. The emergence of ceramide metabolism at twelve weeks does not only suggest an increase in ceramides, but also a progressive impact on sphingolipid biosynthesis, reflecting adaptive or downstream metabolic responses to prolonged treatment. These findings highlight the potential of integrating temporal lipidomic data with the proteomic layer to achieve a comprehensive understanding of drug effects and metabolic adaptations.

In addition, other pathways not strictly related to lipid synthesis and metabolism were observed. In particular, we observed enrichment in genes related to the negative regulation of DNA metabolic process and chromosome organization and condensation at both times (e.g., TERF1, ENPP7, H3C1, and histones H1-5, H1-4, H1-1, and H1-0), while genes enriched in terms related to nucleosome and assembly organization and chromatin organization appeared at the second time point (e.g., H2AX, H2BC9, RBBP4, and H2BC21). These results show potential additional mechanisms that lead lipid-mediated empagliflozin effects. Our method provides directions of research for these results. For example, we can see the signature lipids that led to the selection of gene RBBP4 (UniProt AC Q09028), which ranked better in the second time point than in the first one (compare Supplementary Tables S9 and S10). Of the genes selected among the top 100 at the second time point but not at the first, many are in cluster 11 (chromatin organization; see Supplementary Table S1 and Figure 3C), and RBBP4 is one of them. These results could point to an epigenetic effect of empagliflozin aided by lipids.

Our method points to proteins associated with the lipid signatures using the map geometry, which is itself based on network connectivity. Therefore, it is possible to further substantiate the associations by examining the connections in the network (provided in Supplementary Table S2) with external tools (for example, Cytoscape [62]) to study the paths connecting associated lipids and proteins. For example, in this case study, the protein with the best association score at the second time point (serine palmitoyltransferase 3, UniProt AC Q9NUV7) had already been ranked in the top hundred at the first time point, but then the presence of ceramide (d43:1) (SLM:000391360) in the lipid signature at the second time point pushed it to the top because the protein is directly connected to the lipid by a GWAS interaction [45].

## 4. Discussion and Conclusions

Multi-omics data integration has emerged as a transformative approach to understanding complex molecular mechanisms and their dynamic changes in biological systems. In this study, we present a lipid–metabolite–protein network that leverages hyperbolic mapping to provide an intuitive and biologically meaningful representation of molecular interactions across omics layers. Coupled with a user-friendly software implementation, our framework enhances the ability to explore multi-omics connections and identify biologically relevant associations, with demonstrated utility in literature discovery and functional analysis. The method was implemented as a Shiny application freely available online in a GitHub repository [63]. Users can upload omics datasets (e.g., SwissLipid, UniProt, or PubChem IDs) and obtain ranked results based on hyperbolic distance metrics. The implementation supports interactive processing, detailed result visualization, and downloadable CSV files for further analysis. The software is compatible with R (≥4.0.0) and requires only Shiny and dplyr libraries. Input customization is supported through configurable file paths. The results include ranked lists of related entities and valid subsets for each omics type, presented in an intuitive interface.

### 4.1. Multi-Omics Integration for Disease Insights

Our case study on cardiovascular diseases (CVDs) shows the value of combining different types of biological data with our method. We found lipids and metabolites linked to heart disease-related proteins, many of which are already well-known (like cholesterol and caffeine). We also found less-studied molecules (like 4-imidazoleacetate) that could be new targets for future research. Our method both confirms what we already know and suggests new avenues to explore.

With a second case study, a temporal analysis of empagliflozin’s lipid signatures, we illustrated how to use our method to study how the drug affects the body. Early on, it mainly changes how certain fats (phosphatidylcholine and phosphatidylethanolamine) are processed. Later, it increasingly affects sphingolipid production, specifically increasing sphingomyelins and ceramides. This suggests sphingosine metabolism is key to the drug’s long-term effects. Future research could combine lipid and protein data to see how these changes influence other processes like inflammation, giving us a better understanding of how empagliflozin works.

The two topics used above as examples of application of our approach were specifically chosen because they are related to clinical research that benefits from the determination of functional associations across proteins, lipids, and metabolites. The method can be applied with complete generality to any situation where a researcher has a set of molecules (proteins, lipids, or metabolites) for which similar investigation requirements hold. Naturally, if the input data are enriched in functions and pathways affected in the condition studied, the spatial organization of the map is expected to provide nodes related to similar functions in the other molecular layers. Ultimately, a researcher can examine the network connectivity in detail, for example, to find the connections between a particular protein highlighted by a lipid signature used as input and the lipids in the signature.

### 4.2. Limitations of Our Approach

Our approach is based on the assumption that there is an underlying network of connections among proteins, lipids, and metabolites that is condition-independent, and that parts of this network are activated in different cell types and situations. We are aware that we do not yet fully know this underlying network because there are connections that have not been observed experimentally and because the network does not include all regulatory mechanisms. Regardless, we have shown that it is useful to provide a network linking proteins, lipids, and metabolites, even if limited by incomplete data, as it provides the opportunity to relate expression patterns among these molecular types.

### 4.3. Advantages and Future Directions

We have provided the first method that facilitates analyses seeking to integrate datasets of proteins, lipids, or metabolites, in a transparent manner, using a unique map in which functionally related molecules occupy nearby positions. We have implemented software to use this map with protein, lipid, or metabolite datasets provided by researchers. We have shown that our tool enables biological relevant discoveries when it is necessary to explore connections across these molecular layers, particularly in the context of evaluating experimental data.

Our approach addresses several challenges inherent to multi-omics integration. The use of hyperbolic mapping provides an intuitive way to visualize and interpret complex networks, while the software implementation facilitates the exploration of molecular associations across omics layers. By combining these capabilities, our framework offers a robust platform for studying disease mechanisms and drug effects, and for biomarker discovery.

Unlike frameworks like GLUE [22] or scCross [21], which focus on broad multi-omics alignment (e.g., transcriptomics and chromatin accessibility), our strategy specifically uses enzymatic reactions and genetic associations from domain-specific databases. This bypasses the limitations of methods reliant on paired single-cell data or generic pathway maps, enabling the direct interrogation of biochemical interactions often overlooked in broader analyses, and circumventing alignment challenges posed by non-overlapping molecular layers in conventional multi-omics datasets. On the aspect of enrichment analysis, traditional enrichment tools, like MOPA or ActivePathways, rely on pathway-level statistics or Euclidean distances, which may fail to capture hierarchical dependencies [64,65]. Directional methods, like DPM, penalize inconsistent omics interactions (e.g., inverse mRNA–protein correlations) but lack spatial context. Our approach instead quantifies connectivity through hyperbolic embeddings, enabling nuanced insights into how molecular types interact (e.g., proximity in metabolic networks) rather than whether they align directionally [66].

In our applications illustrating how our method can be used to obtain biological insights, we made some pragmatic choices, such as combining information from three neighbors to score the relationships between different omics layers, or focusing on the top 100 scoring proteins when evaluating the lists of proteins ranked by lipid signatures: our method is flexible in how these thresholds are used, and we envision that different applications might allow exploratory analyses, while others might require statistical support for choosing thresholds. Our method offers the possibility to easily change these thresholds and evaluate the effects on the results. We understand that facilitating the application of our method to different biological and clinical scenarios requires this flexibility and transparency, and with the examples given, we have provided guidelines to achieve this successfully.

Future work could expand our framework by integrating additional regulatory layers, such as miRNAs targeting transcripts, or transcription factor interactions with genes, to capture a more comprehensive view of molecular interactions. Additionally, applying our approach to other disease contexts or therapeutic interventions could validate its generalizability and uncover new biological insights. Finally, incorporating deep learning methods could further improve the performance of our method, as exemplified by other integrative omics approaches, such as CustOmics [16] and scmFormer [17].

In conclusion, our lipid–metabolite–protein network and associated software implementation provide a powerful means for multi-omics integration, offering novel insights into disease biology and drug effects. By bridging the gap between molecular layers, this approach facilitates the discovery of functionally relevant associations, enables hypothesis generation, and supports the identification of potential biomarkers. As multi-omics continues to evolve, frameworks like ours will play a critical role in advancing precision medicine and systems biology.

## Figures and Tables

**Table 1:** Lipids associated with CVD proteins. This table summarizes the results of testing our method using proteins linked to CVD, identifying lipids associated with these conditions. The top four lipids identified through our analysis have been cited more than 600 times in relation to CVD in the scientific literature. The publications were obtained from PubMed using a Boolean search of each lipid in conjunction with the term “cardiovascular disease” (example: “cholesterol esters” AND “cardiovascular disease”).

Lipid	Score	Name	Number of Publications
SLM:000000470	0.01672	Cholesterol esters	184
SLM:000390712	0.01648	Sphingomyelin	121
SLM:000087698	0.01533	Phosphatidylcholine	295
SLM:000489933	0.01520	Ganglioside	15

**Table 2:** Metabolite associations with CVD proteins. This table summarizes the results of testing our method using proteins linked to CVDs, identifying metabolites associated with these conditions. The top ten metabolites identified through our analysis have been cited over 850 times in relation to CVD in the scientific literature. The publications were obtained from PubChem by filtering the literature associated with a given metabolite (in the Literature section of the metabolite record in PubChem) using the search term “cardiovascular disease”.

Metabolite	Score	Name	Number of Publications
Pubchem:800	0.01496	Indoleacetaldehyde	2
Pubchem:751	0.01495	Glyceraldehyde	35
Pubchem:96215	0.01490	4-imidazoleacetate	1
Pubchem:802	0.01437	Indole-3-acetate	24
Pubchem:11850	0.01429	Galactitol	4
Pubchem:5780	0.01393	Sorbitol	46
Pubchem:1150	0.01384	Tryptamine	7
Pubchem:774	0.01382	Histamine	166
Pubchem:2519	0.01358	Caffeine	562
Pubchem:1826	0.01341	5-hydroxyindoleacetate	10

## Data Availability

All data necessary to reproduce our results is provided with this manuscript.

## Supplementary Materials

The following supporting information can be downloaded at https://www.mdpi.com/article/10.3390/biom15040484/s1, Table S1. Nodes of the multi-omics network. Columns indicate the protein, lipid, or metabolite identifiers, the polar coordinates of the node in the map, the cluster number, and whether it was associated with the brain (see Section 2 for details). Table S2. Edges of the multi-omics network. Columns indicate the protein, lipid, or metabolite identifiers, the category of each node, and the database that each edge is obtained from. Table S3. Protein tissue annotations. Columns indicate the protein identifier, the tissue name, and the nTPM. Obtained from [58]. Table S4. Proteins relevant for research on lipids in CVD. UniProt identifiers used as input for the analysis presented in Section 3.2. Literature discovery: Cardiovascular disease case study. Obtained from [43]. Table S5. Lipids scored for associations with CVD proteins. Columns indicate lipid identifiers, scores, and evidence (three closest proteins and distances to them). Table S6. Metabolites scored for associations with CVD proteins. Columns indicate metabolite identifiers, scores, and evidence (three closest proteins and distances to them). Table S7. Lipids dysregulated after one week of treatment with empagliflozin. Used as input in Section 3.3. Functional enrichment analysis of lipid signatures. Columns indicate lipid names and SwissLipids IDs. Obtained from the EmDia study [60,61]. Note that only 9 were in our map (Figure 3B). Table S8. Lipids dysregulated after twelve weeks of treatment with empagliflozin. Used as input in Section 3.3. Functional enrichment analysis of lipid signatures. Columns indicate lipid names and SwissLipids IDs. Obtained from the EmDia study [60,61]. Note that only 3 were in our map (Figure 3C). Table S9. Proteins scored by their associations with lipids dysregulated after one week of treatment with empagliflozin. Columns indicate protein identifiers, scores, and evidence (three closest lipids and distances to them). Table S10. Proteins scored by their associations with lipids dysregulated after twelve weeks of treatment with empagliflozin. Columns indicate: protein identifiers, scores, and evidence (three closest lipids and distances to them). Table S11. Enriched biological processes (GO BP) for lipid signatures at one week after empagliflozin treatment. Columns indicate GO BP terms, overlap (genes in set versus all possible), *p*-values, adjusted *p*-values, odds ratios, combined scores, and genes. Table S12. Enriched biological processes (GO BP) for lipid signatures at twelve weeks after empagliflozin treatment. Columns indicate GO BP terms, overlap (genes in set versus all possible), *p*-values, adjusted *p*-values, odds ratios, combined scores, and genes.
